# Diagnostic Pitfalls in Newborns and Babies with Blisters and Erosions

**DOI:** 10.1155/2009/320403

**Published:** 2010-01-20

**Authors:** Elke Nischler, Alfred Klausegger, Clemens Hüttner, Gabriele Pohla-Gubo, Anja Diem, Johann W. Bauer, Helmut Hintner

**Affiliations:** Department of Dermatology, eb-house Austria, Paracelsus Private Medical University Salzburg, Muellner Hauptstraße 48, 5020 Salzburg, Austria

## Abstract

Establishing the correct diagnosis in newborns presenting with blisters and erosions is not always a straightforward process. Many different disease entities including acquired (i.e., infectious, immunobullous, traumatic) and inherited disorders have to be taken into consideration. Similarities in clinical appearance, colonization and/or superinfections of preexisting skin lesions, as well as the absence of late changes in the neonate often pose significant diagnostic challenges. In this paper we discuss by giving examples the process of making an accurate diagnosis of blistering skin diseases in the neonatal period on the basis of a diagnostic algorithm. In addition, we provide an overview of the rational use and the limitations of laboratory procedures such as microbial testing, routine light microscopy, immunofluorescence antigen mapping, transmission electron microscopy, and molecular genetic analysis.

## 1. Introduction

Neonatal skin differs in structure and function from adult skin, and hence the dermatoses seen during this period differ in their clinical presentation. Newborns are more likely to develop blisters and erosions in response to heat, chemical irritants, and mechanical trauma and are at an increased risk for cutaneous infections [1]. In addition, most hereditary disorders with increased skin fragility may occur first during the neonatal period. Thus, the spectrum of potential differential diagnoses is extensive and ranges from more transient benign to mutilating or potentially life-threatening blistering conditions (Table 1). The distinction between different entities within the first weeks of life is crucial for the further management and the prognosis of the neonate. 

**Figure 1:**
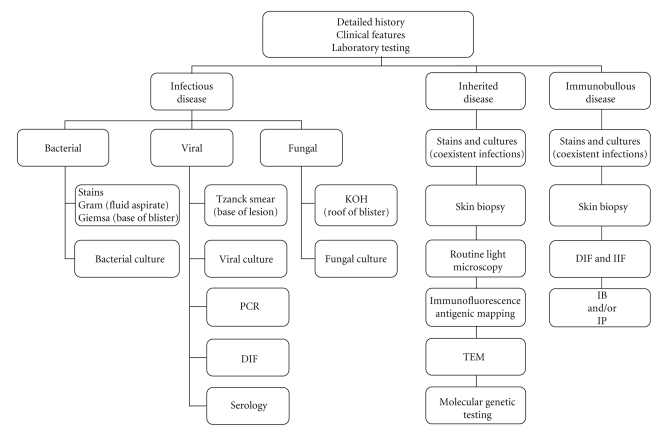
Diagnostic algorithm of blisters and erosions in newborns. DIF: direct immunofluorescence; IB: immunoblot; IIF: indirect immunofluorescence; IP: immunoprecipitation; KOH: potassium hydroxide fungal test; PCR: polymerase chain reaction: TEM: transmission electron microscopy.

In this article we describe common problems (Table 2) in the diagnostic routine on the basis of a report on four selected patients. We recommend emphasizing an algorithmic approach in the diagnostic work up (Figure 1). That includes a detailed medical history (family, maternal, and obstetrical), followed by a complete physical examination (head to toe) and, if needed, an appropriate diagnostic routine such as microbial testing, skin biopsies for histological and ultrastructural assessment, and molecular genetic testing in case of hereditary skin diseases.

## 2. Case Reports

### 2.1. Patient 1—Dominant Dystrophic Epidermolysis Bullosa

**Figure 2:**
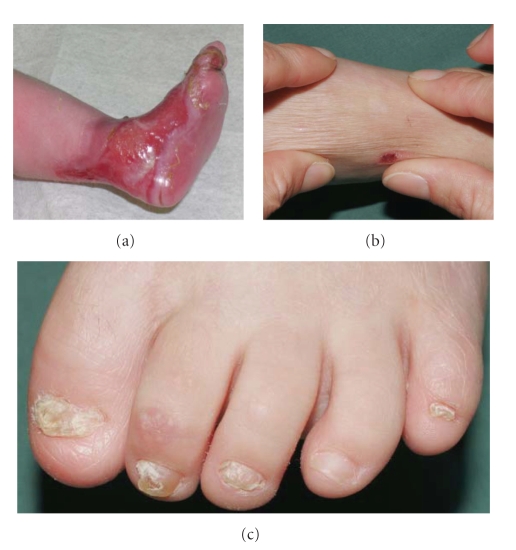
(a) Extensive denuded area enclosing the heel, ankle, joint, and dorsum of the left foot shortly after birth. (b) The same region at the age of 2 years showing an atrophic scar. (c) Dystrophy of toenails.

**Figure 3:**
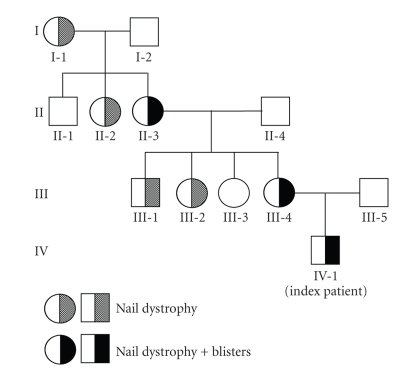
Segregation of the mutation 7120G > A: G2374R and its phenotypic features are shown in the pedigree of the family. The index patient (IV-1) and his grandmother (II-3) show blisters and nail dystrophy. His mother (III-4) presented with blisters solely in childhood and appears with nail dystrophy. All other family members carrying this mutation present with nail dystrophy without blisters.

**Figure 4:**
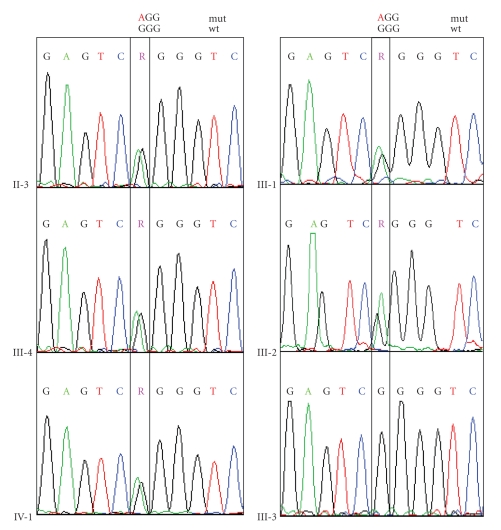
Detection of mutation 7120G > A; G2374R in COL7A1 by sequence analysis in selected family members, except sample III-3, which is wildtype for this locus.

The history of a two-year-old boy revealed that he was born with an extensive skin erosion involving the lower left leg and the dorsum of the left foot (Figure 2(a)). The denuded area reepithelized completely within 3 weeks. Subsequently, small blisters on mechanically strained areas (i.e., hands, knees, and feet) appeared. On clinical examination the two-year-old boy was in good health and presented with tiny erosions on hands and knees, an erythematous plaque with some milia on the left knee, and toenail dystrophy (Figure 2(c)). The lower left leg showed an atrophic scar in the area of the previous erosion (Figure 2(b)). A small, inconspicuous naevus was found above this area. There was a family history of finger- and toenail dystrophy in his mother, maternal aunt and uncle, maternal grandmother and grand aunt, and maternal great grandmother (Figure 3). Besides nail dystrophy the grandmother also showed localized blistering in the pretibial area and the patients mother reported of a period in childhood with mild pretibial blistering. A perilesional skin biopsy of the grandmother, sent to us for immunofluorescence (IF) antigen mapping, showed normal expression patterns of keratin 1, 5, 8, 10, and 14, plectin, bullous pemphigoid antigen-1 (BPAG1) and bullous pemphigoid antigen-2 (BPAG2, also called collagen type XVII collagen), integrins *α*6 and *β*4, laminin 332, and collagen types IV and VII. So far the boy himself was not available for a skin biopsy. Based on the clinical presentation and the family history the diagnosis of dominant dystrophic epidermolysis bullosa (EB) was considered. Mutation detection and screening of the 118 exons of COL7A1 of the index patient was performed using the “priority strategy” and finally confirmed the diagnosis. We could disclose a hitherto unpublished heterozygous mutation defined as transition of G to A at position 7120G > A in exon 93. At the amino acid level this mutation was identified as G2374R. Consequently all available samples of the family members were sequenced forward for this mutation and confirmed reverse on the genetic analyzer ABI 3130 (Figure 4).

Interestingly, the mutation was found in all phenotypes of the disease varying among the affected family members covering blisters and nail dystrophy presented by the index patient and his grandmother. In contrast, his mother had blisters merely in childhood, while many other examined family members showed solely nail dystrophy (Figure 3). 

**Figure 5:**
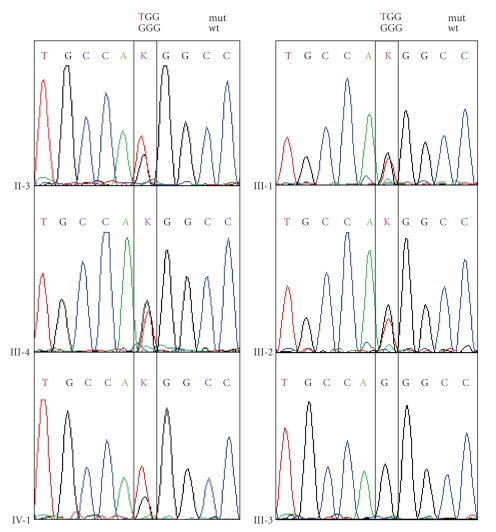
Detection of SNP 7006G > A; G2336W in COL7A1 by sequence analysis in selected family members, except sample III-3, which is wildtype for this locus.

To exclude a possible single nucleotide polymorphism (SNP) we screened 52 wildtype DNA samples for this locus and could confirm only wildtype alleles (not shown). A hithertounpublished heterozygous single nucleotide polymorphism 7006G > T; G2336W was also detected in most of the samples of this family and in one of the screened 50 wildtype samples (Figure 5).

### 2.2. Patient 2—Bullous Impetigo

**Figure 6:**
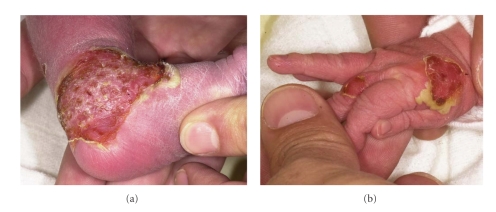
(a) and (b) patient 2: Partially ruptured pustules, resulting in large circumscribed erosions on the right hand and left foot.

A one-day-old, afebril boy was admitted to the neonatal in-patient unit of our hospital. He was born in the fortieth week of gestation after an uneventful pregnancy. Physical findings on admission included pustules and erosions of 1 to 5 cm diameter affecting the feet, the right hand, and the neck (Figures 6(a) and 6(b)). There was no involvement of mucous membranes and the family history did not reveal any bullous skin disorder. Based on the clinical picture the diagnosis of a bullous impetigo was suspected, even though a gram stain of blister fluid was not conclusive. Under systemic antibiotic treatment (augmentin 60 mg/kg bodyweight(BW)/day and netilmicin 6 mg/kg BW/day) marked improvement was observed within 24 hours, with complete healing of the skin lesions by day 7. The subsequent course of the disease (no new blisters occurred) and a positive culture of coagulase positive staphylococcus aureus from a skin swab confirmed the diagnosis of a bullous impetigo. 

### 2.3. Patient 3—Epidermolysis Bullosa Simplex

**Figure 7:**
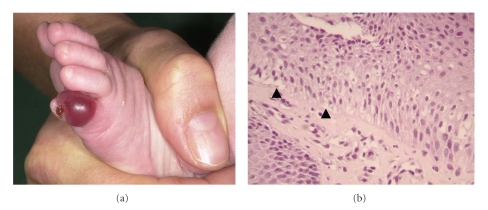
(a) Patient 3: haemorrhagic blister on the left little toe. (b) Histology of perilesional skin shows basal cell cytolysis (arrows) (Hematoxylin-eosin stain; original magnification: X100).

A four-months-old girl was referred to the eb-house Austria for evaluation of a blistering skin disorder. She was the offspring of nonconsanguineous healthy parents and had one older sister who was clinically unaffected. Maternal history revealed recurrent vaginal candida infections during pregnancy. Six days after birth sub- and periungual haemorrhagic blisters on fingers as well as erosions on oral and anal mucosa occurred. Initial and subsequent swabs from the skin and oral mucosa isolated coagulase positive staphylococcus aureus and candida albicans, respectively. Thus the diagnosis of a recurrent bullous impetigo and a soor stomatitis was suspected. Systemic antibiotic (ceftriaxone 50 mg/kg BW/day) and topical antifungal (miconazol nitrate 2% ointment) treatment initially improved the skin and mucous membrane condition, but acral blistering continued to develop (Figure 7(a)). At 4 months of age a skin biopsy of perilesional skin was obtained. Light microscopy studies revealed cytolysis within the epidermal basal cell layer indicating an intrastratum basale split formation (Figure 7(b)). IF antigen mapping confirmed that the level of split formation was through the basal cell layer but showed normal expression of all examined basement membrane proteins (i.e., keratin 1, 5, 8, 10, and 14, plectin, BPAG1 and 2, integrins *α*6 and *β*4, laminin 332, and collagen types IV and VII). The clinical presentation and the detection of an intra-stratum basale split formation led to the diagnosis of an EB simplex. A follow-up examination at 13 months of age revealed small blisters at friction points (i.e., perianal, armpits) and on the palms that healed without scarring.

**Figure 8:**
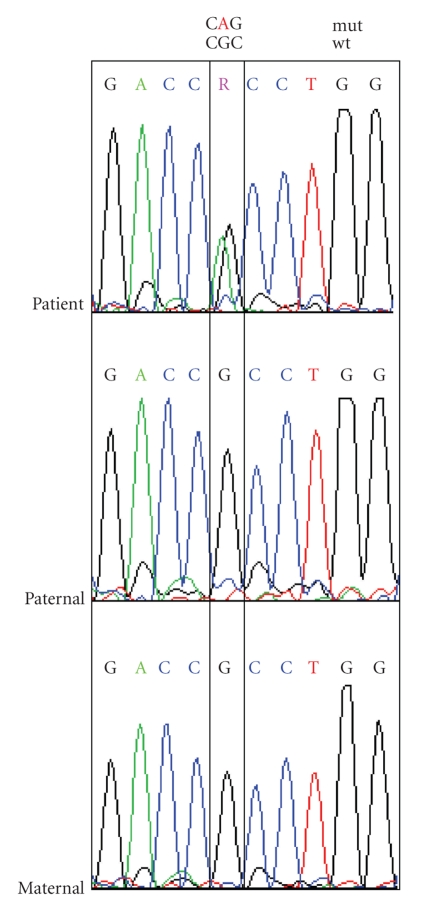
Sequence analysis of 374G > A; R125H in KRT14. The patient is a heterozygous carrier for this *de novo* mutation, whereas the parental alleles reflect wildtype status.

For mutation analysis we screened the genes KRT5 (keratin 5) and KRT14 (keratin 14). To get rid of the pseudogenes in KRT14 a digest of all exons prior to PCR was performed [3]. By sequencing we could identify a well-known [4] and in our case spontaneously arising mutation in KRT14 designated as 374G > A and R125H while the parents were no carriers of this mutation (Figure 8). Stephens et al. [5] had identified codon R125H substitutions in three unrelated sporadic EBS-DM patients which were not found in their clinically unaffected parents. Further de novo cases were published by Shemanko et al. and Müller et al. [6, 7]. This codon is the most commonly affected in all keratin disorders and reflects high mutability. The fact of sporadic arising mutations has therefore implications in future genetic counseling for this family.

### 2.4. Case 4—Recessive Dystrophic Epidermolysis Bullosa

**Figure 9:**
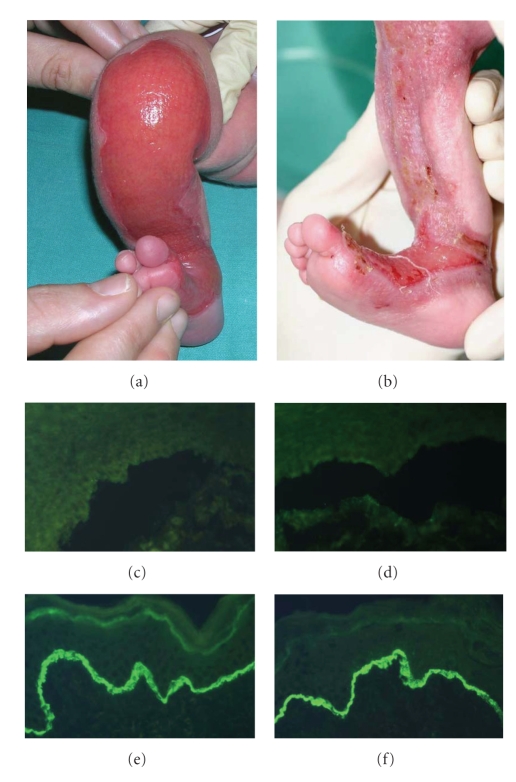
(a) Patient 4: shortly after birth, a large denuded area was found on the left leg. (b) The same leg four weeks later showing healing and scar formation. (c)-(d) IF antigen mapping of perilesional skin: absence of BPAG2 (c) and collagen type VII (d). (e)-(f) IF antigen mapping of clinically unaffected skin (inner aspect upper arm): positive staining for collagen type XVII (e) and collagen type VII (f) ((c)–(f) original magnification: X400).

A girl was born at the thirty-ninth week of gestation with a localized area of absent skin on the right leg and the left sole (demonstrated via teledermatology) (Figure 9(a)). Additionally, a subungual haematoma on the right thumb was present. The girl's older sister was born with a malformation of the cerebellum, but there was no family history for a blistering skin disorder. At one week of age a perilesional skin biopsy was obtained and sent to the eb-house Austria in Michel's transport media [8]. Light microscopy studies of a cryostat section revealed a subepidermal blister formation. IF antigen mapping on perilesional skin showed a positive staining for BPAG1 on the blister roof and for laminin 332 and type IV collagen on the roof and the floor of the split. Interestingly, two major basement membrane proteins, that is, BPAG2 and collagen type VII were absent (Figures 9(c) and 9(d)). A subsequent IF antigen mapping on clinically unaffected skin (inner aspect upper arm) demonstrated bright linear staining of BPAG2 and collagen type VII on the roof of the blister, indicating a dystrophic cleavage (Figures 9(e) and 9(f)). The large skin defects of the lower extremities healed within 6 weeks with atrophic scarring (Figure 9(b)). Subsequently, small blisters appeared in the scar area and on mechanically strained skin and mucosa (i.e., hands, feet, oral and genital mucosa). Additionally, moderate finger- and toenail dystrophy was observed. 

**Figure 10:**
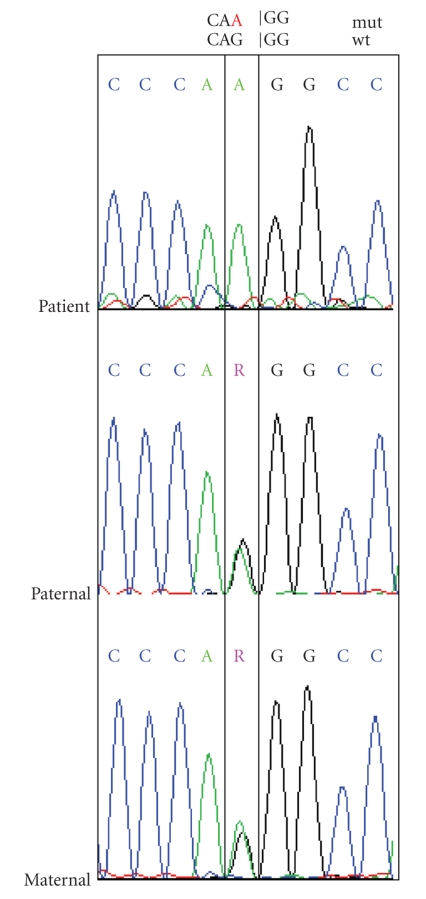
Sequence analysis of 3760-1G > A in COL7A1. The patient is homozygous for this mutation, and parental heterozygous alleles confirm the genetic transmission.

Mutation detection and screening of the 118 exons of COL7A1 gene of the index patient was performed using the “priority strategy.” A novel splice site mutation could be detected in the region of intron 28/exon 29 located as 3760-1G > A in a homozygous status in the patient and was confirmed as heterozygous status in the parents (Figure 10). The mutation is supposed to completely destroy the acceptor splice site. All sequence analyses were done forward and reverse. As we had nonconcordant antigen mapping results for COL7A1 and COL17A1 in two different punch biopsies, we examined DNA of these biopsies for the existence of the splice mutation in COL7A1. The mutation was found homozygous, as expected, in DNA of the first punch biopsy with COL7A1 negative IF, but unexpectedly, it was also found in DNA of the second punch biopsy with COL7A1 positive IF. Further, we screened DNA of the first punch biopsy, which was negative in IF to antibodies against COL17A1, for all COL17A1 exons but we could not disclose any mutation in this gene.

## 3. Discussion

### 3.1. Exact Medical History and Clinical Examination

A detailed family, maternal, and obstetrical history can provide important clues on the nature of the lesions [9]. For example, a maternal history of genital herpes or vaginal yeast infections during pregnancy could point to an infectious etiology (see *patient 3*) whereas a family history of chronic blistering suggests the diagnosis of a hereditary condition. Conversely, skin fragility or trauma-induced blisters may be a minor or even nonexistent feature in some subtypes of EB (i.e., dominant dystrophic EB) [10]. The family history of *patient 1* demonstrates that only the patient, his grandmother, and his mother (exclusively in childhood) showed localized blistering and milia formation in addition to nail dystrophy, whereas in other affected family members nail dystrophy was the only sign of dominant dystrophic EB. Therefore a comprehensive skin examination that includes checking of mucous membranes, hair, and nails should be performed on all family members, not to overlook helpful hints for establishing the correct diagnosis.

### 3.2. Cutaneous Infections

The epidermis, when mature, provides an important barrier against invasion by microorganisms. The maturation (i.e., keratinization) of fetal skin is functionally incomplete at birth and continues over the first few weeks of life [11]. Thus, all neonates are at an increased risk for cutaneous infections that may present as pustules, blisters and/or erosions. Moreover, many infectious agents (i.e., staphylococcus aureus, pseudomonas aeruginosa, and candida species) frequently colonize, and/or superinfect existing erosions and often complicate the diagnostic routine by hiding the underlying pathomechanism [12]. Our findings in *patient 3* illustrate this important point. As EB and bullous impetigo lesions can be very similar in appearance, clinicians should be aware that EB lesions could be coinfected with coagulase positive staphylococcus aureus and take the proper steps to screen for its presence. 

Simple and rapid clinical laboratory tests in the initial work-up for infections are Gram- and Giemsa-stained smears (optimally, fluid from an intact blister/vesicle) and direct microscopy with 10% potassium hydroxide (KOH) preparations [9, 13]. These tests often provide useful information to the clinician regarding presumptive pathogens. However, *patient 2* demonstrates that the Gram stain, like other laboratory tests, has certain inherent limitations and is subject to technical variation and misinterpretation [14]. Therefore, if the initial evaluation does not yield the diagnosis, but an infectious etiology or superinfection is strongly suspected, an appropriate antimicrobial therapy should be introduced and eventually changed depending on the results of confirmatory tests such as bacterial, fungal, and viral cultures, serology, and PCR. The prompt recognition and appropriate treatment of cutaneous infectious diseases in neonates is essential to shorten the course of the disease as well as to prevent widespread dissemination, particularly in patients with a disrupted skin barrier. Moreover for contagious skin diseases such as bullous impetigo appropriate hygienic measures are important to prevent spread to others. If skin lesions do not clear under appropriate antimicrobial treatment and/or new blisters continue to develop, a skin biopsy should be taken.

### 3.3. The Skin Biopsy as Standard for Diagnosis

Although it is an invasive procedure, one should not hesitate with the decision for a skin biopsy on neonates. Histological and ultrastructural assessment of skin samples can often facilitate and accelerate the proof or exclusion of various differential diagnoses [15]. If a hereditary cause is suspected, the biopsy should be taken from clinically normal appearing skin. It may be helpful to induce a blister by slightly rotating a pencil eraser at the site of the biopsy [16]. The purpose of taking a biopsy from clinically normal skin is to avoid the appearance of secondary changes. For example, subepidermal blisters may appear to be intraepidermal if regeneration of new epithelium occurs in an older lesion [17]. Additionally, older lesions that contain necrotic tissue tend to accumulate neutrophils and proteolytic degradation of basement antigens can influence the outcome of IF examinations [18]. 

*Patient 4* with recessive dystrophic EB illustrates this important point. While IF analysis of perilesional skin revealed absence of *collagen type VII* and *BPAG2*, both proteins could be detected in unaffected skin. These findings suggest a proteolytic degradation of both proteins in perilesional/lesional skin.

**Routine Light Microscopy**

Allows the pathologist to identify the anatomical level of the split (i.e., subcorneal, suprabasal, “subepidermal”) and the nature of the inflammatory infiltrate in the dermis or within the blister [19]. For example, histologic features of the blisters in bullous impetigo include loss of cell adhesion in the superficial epidermis, just below the stratum corneum and an inflammatory infiltrate, mostly consisting of neutrophils, whereas inherited EB usually presents as subepidermal bullae without inflammatory cells [20, 21]. However, in some cases of mainly newborn EB patients there is a predominantly eosinophilic infiltrate [22]. The appearance of a significant number of eosinophils associated with a subepidermal blister formation might suggest blistering diseases caused by immunologically mediated cleavage such as bullous pemphigoid or linear IgA dermatosis. These autoimmune blistering diseases can be ruled out by direct IF examinations that detect immune deposits in the epidermal basement membrane zone. Routine light microscopy is generally not very helpful in delineating a specific subtype of EB, since both junctional and dystrophic subtypes represent with “subepidermal” splits [2, 23]. Therefore precise classification of individual patients with EB requires determination of the level of tissue separation by IF antigen mapping and/or transmission electron microscopy.

**Immunofluorescence Antigen Mapping and/or Transmission Electron Microscopy (TEM)**

IF antigen mapping is a modified IF technique whereby the ultrastructural level of skin cleavage can be deduced by determination of the localization (i.e., roof versus floor of an induced blister) of binding within an EB skin specimen of a series of antibodies having known ultrastructural binding sites [24, 25]. For example, anti-BPAG2 (type XVII collagen) antibody can be used to stain the lower surface of basal keratinocytes, and antitype IV collagen antibodies will stain the lamina densa [2, 24–26]. In the simplex variants of EB with an intraepidermal separation at the level of basal cells all antibodies will stain the floor of the split. In junctional types of EB the separation occurs within the lamina lucida and therefore antibodies against BPAG2 will bind on the roof of the blister whereas antitype IV collagen antibodies stain the floor. In dystrophic forms of EB, where the separation occurs beneath the lamina densa, all antibodies will stain the roof of the split [2, 27]. Additional studies with a selected series of antibodies (i.e., in addition to the above mentioned, keratin 5 and 14, plectin, integrins *α*6 and *β*4, laminin 332, BPAG2, and type VII collagen) may reveal defective or the lack of proteins and therefore may provide clues concerning the genetic origin [23, 28]. Besides IF antigen mapping, TEM is another major diagnostic technique for the diagnosis of EB. The primary advantage that TEM has over IF antigen mapping is that it is the only technique whereby associated ultrastructural findings can be directly assessed and quantified [15]. More specifically, direct assessment of possible morphologic or morphometric alterations of some cellular or basement membrane-associated structures (i.e., hemidesmosomes with subbasal dense plates, anchoring filaments or fibrils, and tonofilaments) may be of diagnostic importance for specific subtypes of EB [29, 30]. However, TEM is a relatively expensive and time consuming technique and the accurate interpretation of the above described studies demands considerable experience on the part of the pathologist. Whereas TEM and IF antigen mappings were thought to have equal sensitivity and specificity, a recent comparative study between these two techniques demonstrated that IF antigen mapping may be a more accurate diagnostic test for the major types of EB [31].

### 3.4. Molecular Genetic Testing

The importance of an accurate molecular diagnosis for patients with heritable blistering diseases should not be underestimated since it allows better prognostication regarding the severity and prediction of the progress of the disease [32]. Moreover the identification of specific mutations has profound implications for genetic counseling of families at risk for recurrence of the disease in the same and subsequent generations. An example is provided by our* patient 4* with a relatively mild dystrophic form of EB and no family history. The clinical manifestations could result either from a de novo dominant mutation (dominant dystrophic EB) in one of the COL7A1 allels or from a recessive mutation (recessive dystrophic EB) in both COL7A1 allels [33]. These two possibilities are only distinguishable by mutation analysis [34]. In our patient identification of 2 mutant alleles in the proband's DNA and demonstration of their inheritance from the respective parents led to the diagnosis of a recessive dystrophic EB. Further implications are that the risk of our patient having an affected offspring is very low owing to relatively low carrier frequency of the corresponding gene mutations in the general population, while the risk for the parents of having another affected offspring is 1 in 4, or 25% [34, 35].

Furthermore, molecular testing is the basis for future development of gene therapy and other novel treatment modalities. However, considering the expense and labor-intensiveness of this approach, molecular testing still remains primarily a research tool for the postnatal diagnosis. The impact of molecular genetics is the most evident in prenatal testing, that has proved to be of great benefit for couples in which one or both partners are patients, or couples who have already had one affected pregnancy [36]. An extension of prenatal testing is the development of preimplantation genetic diagnosis, a technique licensed for severe forms of EB [36].

## 4. Conclusion

Our observations highlight simple pitfalls in establishing a correct diagnosis of blistering skin diseases in newborns. Discordance among different assessments and clinical findings should prompt reassessment of the diagnosis in search of potential pitfalls. In confusing situations, or when hereditary or immunobullous causes are considered, we suggest referring the neonates to a center such as the eb-house Austria that specializes in bullous skin diseases. A skin biopsy within the first week of life and subsequent histopathological, immunohistochemical, and ultrastructural analysis can narrow the spectrum of potential differential diagnoses. In case of hereditary skin diseases detection of gene mutations can confirm the clinically and microscopically suspected diagnosis and is the basis for genetic counseling and prenatal diagnosis.

## Figures and Tables

**Table 1:** Differential diagnosis of erosions and blisters in the neonate and young *c* *h* *i* *l* d*.

Inherited or congenital disorders	Acquired disorders
Epidermolysis bullosa hereditaria—all types (simplex, junctional, dystrophic)	*Immunobullous disorders*
Ichthyosis bullosa of Siemens	Epidermolysis bullosa acquisita
Netherton syndrome	Linear IgA dermatosis
Congenital peeling skin syndromes	Bullous pemphigoid
Pachyonychia congenita	Cicatricial pemphigoid
Kindler's syndrome	Pemphigus
Congenital porphyria	*Infectious diseases*
Acrodermatitis enteropathica	Herpes simplex
Incontinentia pigmenti	Bullous impetigo
Ectodermal dysplasia	Staphylococcal scalded skin syndrome
AEC syndrome (Hay-Wells syndrome)	Congenital lues (pemphigus syphiliticus)
Ectodermal dysplasia with plakophilin 1 deficiency	Other bacterial, viral or fungal infections
Congenital absence of skin (cutis aplasia)	*Other diseases or conditions*
Congenital erosive dermatosis with reticulate supple scarring	Bullous mastocytosis
Mendes da Costa syndrome	Behcet disease
Shabbir's syndrome (laryngo-onychocutaneous syndrome)	Traumatic blisters (sucking, other)
Epidermolytic hyperkeratosis (bullous ichthyosiform erythroderma)	Toxic epidermal necrolysis

*Modified by Eady et al. [2].

**Table 2:** Variables causing problems in differential diagnosis.

*Clinical features*
Similarity and overlapping of clinical features
Coexistance of different diseases
Colonization and/or superinfection
Absence of late changes in the neonate (scar formation, pigmentation changes, nail dystrophy)
*Diagnostical methods *
Sample (site of sample not representative, secondary alterations)
Timepoint of sample collection (i.e., higher herpes simplex virus load in early vesicles)
Sensitivity and specifity of method
Technical problems (wrong transportation media,…)
Experience in interpreting the results

## Abbreviations (in alphabetical order)

BPAG: Bullous pemphigoid antigen
DIF: Direct immunofluorescence
EB: Epidermolysis bullosa
EBS-DM: Epidermolysis bullosa simplex Dowling-Meara
IB: Immunoblot
IF: Immunofluorescence
IIF: Indirect immunofluorescence
IP: Immunoprecipitation
KOH: Potassium hydroxide
KRT: Keratin
PCR: Polymerase chain reaction
SNP: Single nucleotide polymorphism
TEM: Transmission electron microscopy.
